# Associations between cooking fuel use, its transitions, and worsening sensory impairments among Chinese middle-aged and older adults: a cohort study

**DOI:** 10.1186/s12877-024-04746-3

**Published:** 2024-03-27

**Authors:** Shaojie Li, Guanghui Cui, Mingzheng Hu, Yang Hu, Longbing Ren, Yuling Jiang, Jing Sun, Zhe Luan, Kejia Hu, Yunquan Zhang, Gang Sun, Yao Yao

**Affiliations:** 1 School of Public Health, Peking University, Beijing, China; 2 China Center for Health Development Studies, Peking University, Beijing, China; 3 Department of Integrated Traditional Chinese and Western Medicine, Peking University First Hospital, Beijing, China; 4 Department of Community Nursing, School of Nursing, Peking University, Beijing, China; 5 Department of Gastroenterology and Hepatology, First Medical Center of the PLA General Hospital, Beijing, China; 6 Department of Big Data in Health Science, School of Public Health, Key Laboratory of Intelligent Preventive Medicine of Zhejiang Province, Zhejiang University, Hangzhou, China; 7 Department of Epidemiology and Biostatistics, School of Public Health, Hubei Province Key Laboratory of Occupational Hazard Identification and Control, Wuhan University of Science and Technology, Wuhan, China

**Keywords:** Household air pollution, Sensory impairments, Cooking fuels, China

## Abstract

**Background:**

This study aimed to explore the associations between household air pollution (HAP), measured by cooking fuel use, sensory impairments (SI), and their transitions in Chinese middle-aged and older adults.

**Methods:**

Participants were recruited from the 2011 China Health and Retirement Longitudinal Study (CHARLS) and were subsequently followed up until 2018. Data on SI were collected by self-reported hearing and vision impairments, which were divided into three categories: non-SI, single SI (hearing or vision impairment), and dual SI (DSI). Cooking fuels, including solid and clean fuels, are proxies for HAP. The transitions of cooking fuels and SI refer to the switching of the fuel type or SI status from baseline to follow-up. Cox proportional hazard regression models were used to explore associations, and hazard ratios (HRs) and 95% confidence intervals (CI) were used to evaluate the strength of the association.

**Results:**

The prevalence of non-SI, single SI, and DSI was 59.6%, 31.8%, and 8.6%, respectively, among the 15,643 participants at baseline in this study. Over a median follow-up of 7.0 years, 5,223 worsening SI transitions were observed. In the fully adjusted model, solid fuel use for cooking was associated with a higher risk of worsening SI transitions, including from non-SI to single SI (HR = 1.08, 95% CI = 1.01–1.16) and from non-SI to DSI (HR = 1.26, 95% CI = 1.09–1.47), but not from single SI to DSI. In addition, compared to those who always used solid fuels, participants who switched from solid to clean fuel for cooking appeared to have attenuated the risk of worsening SI transitions. The statistical significance of the associations remained in the set of sensitivity analyses.

**Conclusion:**

Solid fuel use was associated with higher risks of worsening SI transitions, while converting the type of cooking fuel from solid to clean fuels may reduce the risk of worsening SI transitions. Our study suggests that tailored clean fuel interventions, especially in developing countries, should be implemented to prevent sensory impairments and hence reduce the burden of sensory impairment-related disability.

**Supplementary Information:**

The online version contains supplementary material available at 10.1186/s12877-024-04746-3.

## Background

Sensory impairments (SI) refer to the functional loss or decline of a person’s perception of vision, hearing, smell, and other senses due to physiological disorders, diseases, and age [1, 2], which include vision impairment (VI), hearing impairment (HI), etc. According to the World Hearing and Vision Report released by the World Health Organization in 2019, approximately 29% had VI and 20% had HI among the 7.5 billion people worldwide [3, 4]. As the population ages, the prevalence of dual sensory impairments (DSI; both hearing and vision impairments coexist) will continue to increase [5]. Previous large observational studies have established that SI is associated with an increased risk of multiple adverse health outcomes such as cognitive impairment [6], dementia [7], and mortality [8].

Given the high prevalence and adverse health effects of SI, many studies have explored modifiable risk factors for SI, such as sociodemographic characteristics, lifestyles, and disease-related factors [9, 10]. However, few studies have focused on environmental factors, especially the relationship between the household environment and SI. Household air pollution (HAP) is an indoor environmental factor that affects health and is defined by the WHO as a primary reliance on solid fuels such as wood, crop residue, coal, or dung for cooking, heating, and lighting [11]. Solid fuel use produces particulate matter (PM) with a diameter of less than 2.5 μm (PM_2.5_), which diffuses into the home, causing HAP and ultimately endangering the health of every member of the family [12]. There are distinct regional differences in global solid fuel use. A previous study of 98 low- and middle-income countries (LMICs) showed that the use rate of solid fuels reached 56.5% in 2018 [13]. China is among the LMICs with the largest number of people cooking solid fuels. A previous large epidemiological study found that nearly half of adults over the age of 50 in China (47.5%) used solid fuels for cooking [14]. The association between HAP caused by cooking fuels and adverse health outcomes has been demonstrated in numerous studies [15].

Previous studies have found that PM emitted from indoor solid fuel use can cause oxidative damage to human alveolar epithelial cells and increase the level of inflammatory factors, thereby inducing the occurrence of a variety of diseases [16]. It is worth noting that inflammatory factors and oxidative stress canpromote retinal degeneration by inducing senescence in retinal pigmented epithelial cells and accelerate auditory neuron loss [17, 18]. Given the common pathway involved in the two, HAP from cooking fuels may be the cause of SI. Research on Korean adults has found that long-term exposure to outdoor air pollutants is associated with an increased risk of hearing loss [19]. A cross-sectional study of children in China found that air pollution was associated with a higher risk of vision impairment [20]. Previous studies have shown that indoor air pollutants escape to the outside, which is the main source of fine particulate air pollution in the outdoor environment of LMICs, and generally contribute more than traffic, industry, and power generation [21]. This means that HAP may be more harmful to sensory function than outdoor air pollution. Thus, it can be inferred that HAP from cooking fuels may be associated with a higher risk of SI. Several previous studies have explored the association between cooking fuel and a single SI, such as VI [22, 23] and hearing loss [24]. However, it is important to mention that most existing studies focus on a single SI, and it is not clear whether HAP is associated with DSI. Previous studies have confirmed a stronger association between DSI and adverse health outcomes, such as dementia [7] and all-cause mortality [25], compared with a single SI. Moreover, few studies have focused on the association between cooking fuel and SI transition.

Therefore, to fill the gaps in the literature mentioned above, this study aimed to explore the associations between cooking fuel, SI, and their transitions in middle-aged and older Chinese adults.

## Methods

### Participants

**Fig. 1:**
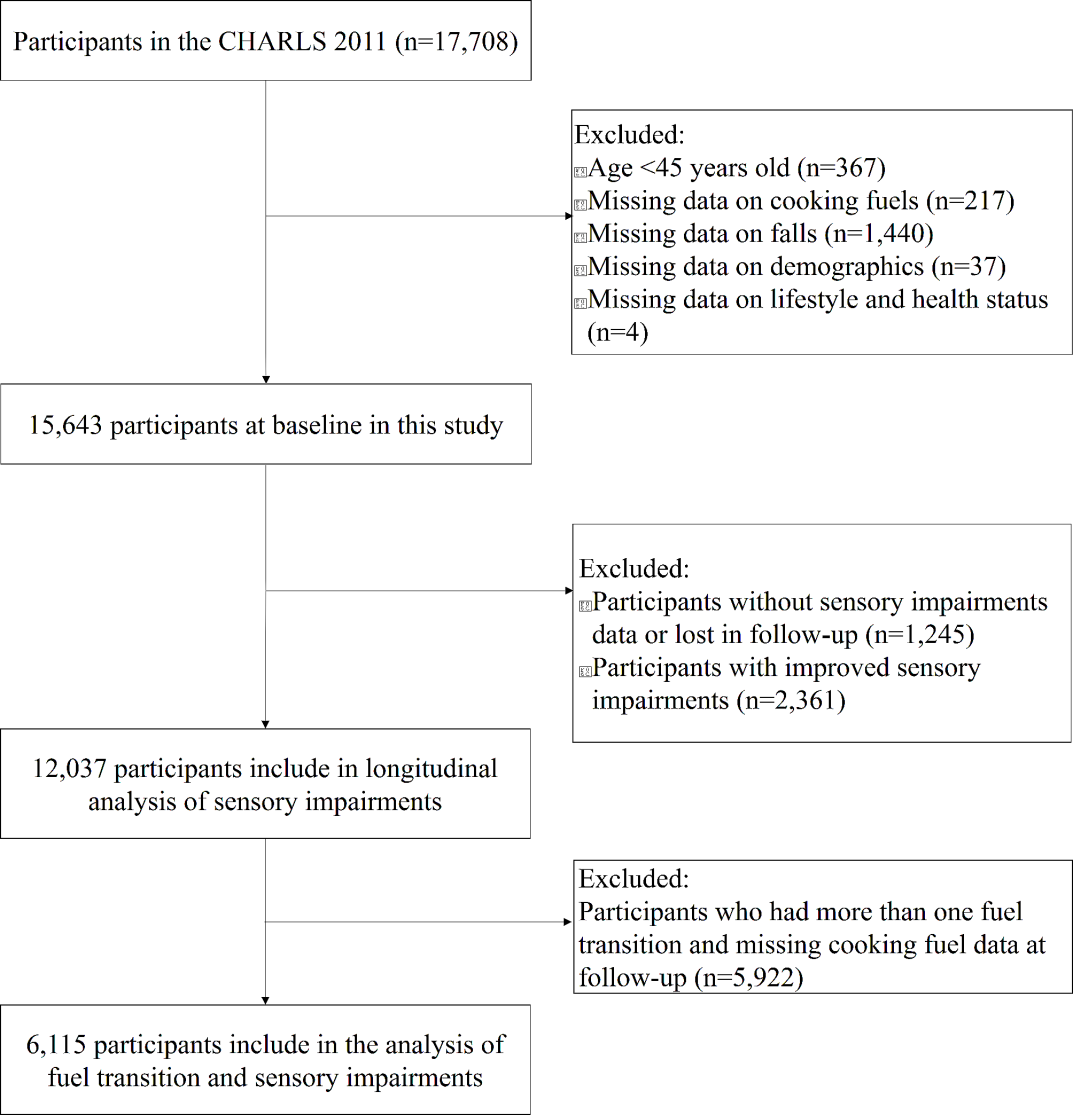
The selection process of participants in this study

Data for this study were obtained from a nationally representative aging survey, the China Health and Retirement Longitudinal Study (CHARLS, 2011–2018 wave). The details of CHARLS have been reported in previous studies [26]. First, we used the CHARLS 2011 wave as a baseline survey and followed it up in 2013, 2015, and 2018, respectively. The inclusion criterion for this study was age ≥ 45 years. The exclusion criteria were missing data on key variables such as SI, cooking fuels, age, sex, and residence. In 2011, the CHARLS recruited 17,708 participants. After screening out participants aged < 45 years and those with missing information on key variables, 15,643 participants were included at baseline. In the longitudinal analysis, we excluded participants with improved SI from baseline to follow-up (because SI is a degenerative disease, its improvement is mostly due to medical intervention, such as wearing hearing aids or glasses), those who were lost to follow-up, and missing SI data. Finally, 12,037 participants were included in the analysis of SI transitions. It is important to mention that, referring to a previous study on cooking fuel transition [27], we excluded participants who had more than one fuel transition and missing cooking fuel data at follow-up. Finally, 6,115 participants were included in the analysis of association between the cooking fuel transition and SI. The selection process of the participants in this study is shown in Figure 1. CHARLS has been approved by the Biomedical Ethics Review Committee of Peking University, and written informed consent was obtained from all participants.

### Measures

#### HAP

In the CHARLS, participants were asked about the main fuel for cooking at baseline and follow-up. Coal, crop residue, and wood burning are classified as solid fuels, while natural gas, marsh gas, liquefied petroleum gas, and electricity are classified as clean fuels. Based on the cooking fuels asked in the four surveys, we divided the cooking fuel transition into four groups: always solid fuel, clean to solid fuel, solid to clean fuel, and always clean fuel.

## SI

SI was assessed using self-reported HI and VI. In CHARLS, for HI, participants were asked, ‘How is your hearing?’. The answers were excellent, very good, good, fair, and poor. A response to the question as “poor” was judged as HI. VI was evaluated using both long-distance and near vision. Participants were asked how well they looked at distant and near vision, and the answers were the same as for HI, that is, excellent, very good, good, fair, or poor. A response of either of the two questions as “poor” was judged as VI. The evaluation methods for SI mentioned above have been widely used in previous studies [28, 29]. When HI and VI coexist, they are determined as a DSI. In this study, SI was divided into three categories: non-SI, single SI (hearing or vision impairment), and DSI. As mentioned in the participants’ section, since SI is unlikely to show improvement on the basis of not implementing medical interventions [30, 31], to avoid confusion, this study only focused on the worsening transition of SI. In this study, we mainly focused on three types of SI worsening transitions: non-SI to single SI, non-SI to DSI, and single SI to DSI.

### Covariate

In this study, we set sociodemographic characteristics, lifestyles, and chronic diseases as potential covariates, including age, sex (male vs. female), residence (urban vs. rural), marital status (married vs. unmarried), educational level (middle school below vs. middle school and above), family wealth (low vs. high), smoking (no vs. yes), drinking (no vs. yes), exercise (hardly vs. regularly), and self-reported chronic diseases diagnosed by a doctor (no vs. yes).

### Data Analysis

Continuous variables are described as mean ± standard deviation. Categorical variables are presented as n (%). Cox proportional hazard regression models were used to explore the associations between cooking fuels, SI, and their transitions in the longitudinal analyses based on the adjustment of all covariates. To test the robustness of the above associations, we conducted two sensitivity analyses. Specifically, we excluded participants who may have mild cognitive impairment (MCI) and then conducted cox model because they may have a heavier recall bias when reporting cooking fuels or SI. In the second sensitivity analysis, we further included the outdoor annual mean PM_2.5_ concentration as a covariate because a previous study found that outdoor air pollution may be associated with SI [32]. The measurement details of MCI and PM_2.5_ are displayed in the Supplementary Materials. Hazard ratios (HRs) and 95% confidence intervals (CIs) were calculated to assess the associations between cooking fuels, SI, and their transitions. We performed all analyses using STATA 17.0 (Stata Corp, College Station, TX, USA), and *P* < 0.05 indicated statistical significance.

## Results

### Descriptive statistics

The characteristics of the participants according to the cooking fuel are shown in Table 1. A total of 15,643 middle-aged and older adults were included at baseline in this study. The mean age of the participants in this study was 59.4 ± 9.7 years. At the baseline, the utilization rates of solid fuel and clean fuel for cooking were 53.8% and 46.2%, respectively. The prevalence of non-SI, single SI, and DSI was 59.6%, 31.8%, and 8.6%, respectively, at baseline.

**Table 1:** Characteristics of participants according to HAP at baseline

Variables	CHARLS		
	Total sample	Clean fuel	Solid fuel
Total sample	15,643 (100.0)	7230 (46.2)	8413 (53.8)
Age, years			
45–59	8632 (55.2)	4341 (60.0)	4291 (51.0)
≥ 60	7011 (44.8)	2889 (40.0)	4122 (49.0)
Sex			
Male	7454 (47.7)	3437 (47.5)	4017 (47.7)
Female	8189 (52.3)	3793 (52.5)	4396 (52.3)
Residence			
Rural	11,993 (76.7)	4093 (56.6)	7900 (93.9)
Urban	3650 (23.3)	3137 (43.4)	513 (6.1)
Marital status			
Married	13,629 (87.1)	6340 (87.7)	7289 (86.6)
Unmarried	2014 (12.9)	890 (12.3)	1124 (13.4)
Educational level			
Middle school below	10,472 (66.9)	3929 (54.3)	6543 (77.8)
Middle school and above	5171 (33.1)	3301 (45.7)	1870 (22.2)
Family wealth			
Low	7674 (49.1)	3288 (45.5)	4386 (52.1)
High	7969 (50.9)	3942 (54.5)	4027 (47.9)
Smoking			
No	9491 (60.7)	4527 (62.6)	4964 (59.0)
Yes	6152 (39.3)	2703 (37.4)	3449 (41.0)
Drinking			
No	10,547 (67.4)	4822 (66.7)	5725 (68.0)
Yes	5096 (32.6)	2408 (33.3)	2688 (32.0)
Exercise			
Hardly	9101 (58.2)	3933 (54.4)	5168 (61.4)
Regularly	6542 (41.8)	3297 (45.6)	3245 (38.6)
Chronic diseases			
No	4989 (31.9)	2430 (33.6)	2559 (30.4)
Yes	10,654 (68.1)	4800 (66.4)	5854 (69.6)
Hearing impairment			
No	13,443 (85.9)	6488 (89.7)	6955 (82.7)
Yes	2200 (14.1)	742 (10.3)	1458 (17.3)
Vision impairment			
No	10,181 (65.1)	5107 (70.6)	5074 (60.3)
Yes	5462 (34.9)	2123 (29.4)	3339 (39.7)
Sensory impairment			
No	9324 (59.6)	4772 (66.0)	4552 (54.1)
Single	4976 (31.8)	2051 (28.4)	2925 (34.8)
Dual	1343 (8.6)	407 (5.6)	936 (11.1)

### Association analyses of cooking fuels, SI and their transitions

**Fig. 2:**
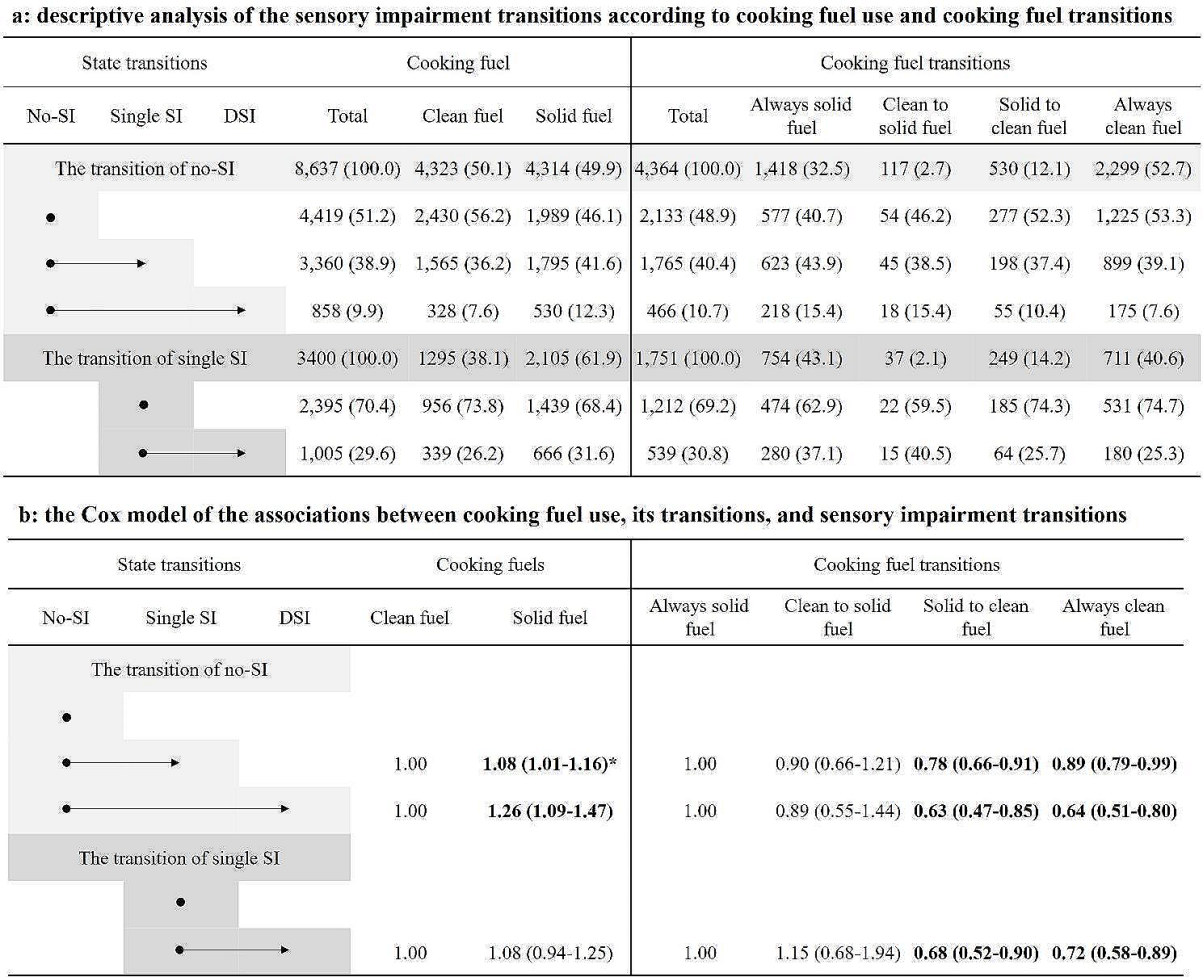
Descriptive and association analyses of cooking fuels, transitions and sensory impairment transitions Note: Bold refers to statistical significance in b. *: hazard ratio (95% confidence interval) (all such values)

Figure 2 shows the results of the descriptive and association analyses of the cooking fuels, SI, and their transitions. Specifically, over a median follow-up of 7.0 years, 5,223 worsening SI transitions were observed. In the fully adjusted model, solid fuel use for cooking was associated with higher risks of worsening SI transitions, including from non-SI to single SI (HR = 1.08, 95% CI = 1.01–1.16) and from non-SI to DSI (HR = 1.26, 95% CI = 1.09–1.47), but not from single SI to DSI. In addition, we also found that compared with those who always used solid fuels, participants who switched from solid to clean fuel for cooking appeared to have attenuated risks of all worsening SI transitions, including from non-SI to single SI (HR = 0.78, 95% CI = 0.66–0.91), from non-SI to DSI (HR = 0.63, 95% CI = 0.47–0.85), and from single SI to DSI (HR = 0.68, 95% CI = 0.52–0.90). However, we did not find a significant association between the conversion from clean fuel to solid fuel and worsening SI transition.

### Sensitivity analysis

**Fig. 3:**
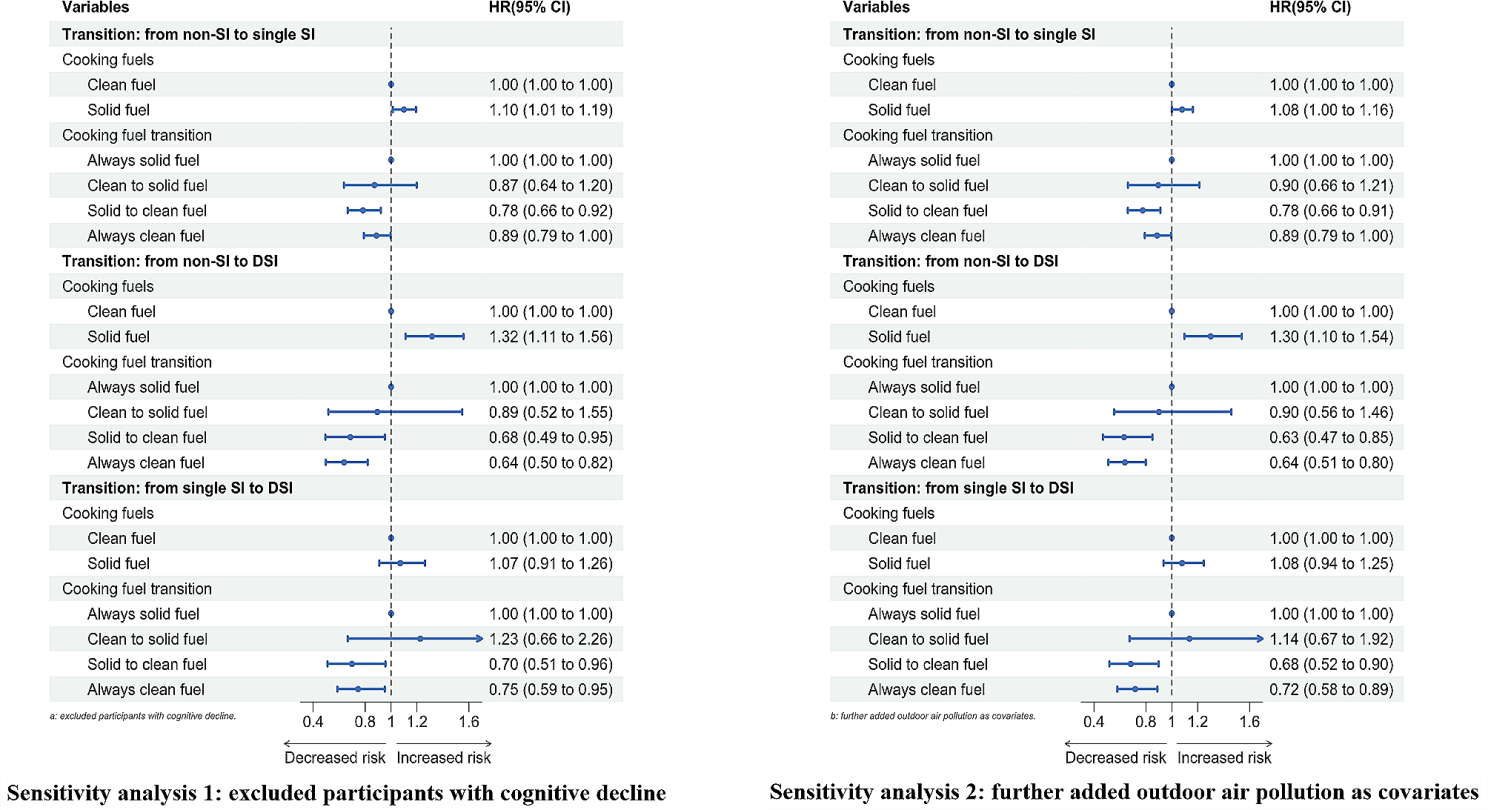
Sensitivity analysis

In the sensitivity analysis (Fig. 3), we excluded participants with MCI and repeated all analyses. The results showed that the associations of cooking fuels and their transition with worsening SI transitions were also statistically significant. In addition, considering the possible confounding caused by outdoor air pollution, we further adjusted for the annual average PM_2.5_, and the results showed that solid fuel use for cooking and the transitions of cooking fuels were also significantly associated with worsening SI transitions in sensitivity analysis 2. Sensitivity analyses showed that these associations were robust.

## Discussion

In this study, we used nationally representative data on middle-aged and older adults from China to explore the associations between cooking fuels, SI, and their transitions. We found that solid fuel use, which often refers to exposure to HAP, was associated with a higher risk of worsening SI transitions. In addition, compared to those who always used solid fuels, participants who switched from solid to clean fuel for cooking appeared to have attenuated the risk of worsening SI transitions. Our findings increase our understanding of the potential adverse effects of cooking fuels on SI.

The first important finding of our study is that solid fuel use for cooking is associated with an increased risk of two worsening SI transitions: from non-SI to single SI and from non-SI to DSI. A recent cohort study found that cooking with biomass fuels was associated with a greater risk of VI among older Chinese adults [22], which is similar to our study. In addition, a cross-sectional study in India found that the use of unclean cooking fuels was related to VI in older adults [23]. A recent study also found that household solid fuel use is associated with an increased risk of hearing loss in the Chinese population [24]. These studies provide evidence that supports our findings. In addition, our study further expanded the association between HAP and SI, that is, worsening SI transition. The association between cooking fuels and worsening SI transitions may involve mechanisms such as inflammatory responses, oxidative stress, and immune responses. Previous studies have found that environmental PM produced by HAP can cause oxidative stress and a decrease in endogenous antioxidants [33], which can induce an inflammatory response and produce proinflammatory cytokines such as IL-1β [34]. In addition, air pollutants may also lead to impaired macrophage function owing to overload and interference with antimicrobial immune processes in vitro, causing immune dysregulation [35]. Damaged macrophages, increased levels of inflammatory factors, and oxidative stress in the body may cause damage, degeneration, or loss of cochlear hair cells or spiral ganglion neurons in the auditory system [36, 37] and enhance ocular surface and retinal inflammation to increase the risk of VI [38]. These physiological studies suggest that HAP may persistently impair sensory function, thereby increasing the risk of worsening SI transition.

Another important finding of this study is that the transition from solid to clean fuel is associated with a lower risk of worsening SI transition. Although a previous study also explored the association between fuel switching and VI in older adults, no significant association was found between switching from biomass to clean fuel and VI [22]. This may be related to different sample sources and cohort follow-up times. The mean age of the study population in the above study was 82.6 years [22], which is significantly higher than that of our study population (59.4 years). In China, people over 80 years of age may not cook for themselves due to their reduced self-care ability [39], thus making exposure to solid fuels less hazardous. In addition, the follow-up period of our study (7 years) was significantly longer than that of the aforementioned study (2 years) [22]. These differences in study design may account for the different results. In addition, our study further extends previous findings, as we focus not on a particular SI, but on SI transitions. Our research showed that switching from solid to clean fuels can reduce the risk not only from non-SI to single SI and DSI but also from single SI to DSI. This finding provides solid evidence for future interventions for SI from a clean energy promotion perspective. Previous observational studies have also provided preliminary insights into the potential health benefits of cooking fuel transitions [27, 40]. For example, a study based on the China Health and Nutrition Survey found that switching from polluting to clean fuel prevented the risk of hypertension [27]. A previous cohort study also found that the transition from polluting to clean cooking fuels is associated with reduced excess deaths in China [40]. However, it is important to mention that we did not find a significant association between the transition from clean to solid fuels and worsening SI transitions, probably due to its low percentage (< 3%) and small sample size. Another possible explanation is that health hazards due to solid fuel use are long-term effects that may not be detectable in the short-term. In the CHARLS survey, we did not ask participants in detail about the reasons and times for the transitions of cooking fuel, which further limited our interpretation. We speculate that the transition from clean to solid fuels may be a reflection of a decline in the economic level of the participants’ households, which may have health hazards, but this result was not found due to the limitations of the sample size and uncollected fuel transition time. This also suggests the need to further explore this issue in future studies with larger sample sizes.

This study has important implications for the current prevention and intervention efforts for SI in middle-aged and older adults. Over the past 20 years, the Chinese government has vigorously implemented several clean energy programs, such as coal-to-gas conversion and universal access to electricity [41, 42]. However, due to economic inequality, people in many low-income regions continue to use solid fuels for cooking. Considering China’s increasing aging trend, this study suggests that the government should further promote clean energy penetration, which may help reduce the incidence of SI in middle-aged and older adults and promote healthy aging. For example, the government can promote the addition of piped natural gas in rural areas and older urban neighborhoods, establish special subsidies for natural gas installation and use, expand the scale of natural gas use, and promote the large-scale use of clean fuels such as natural gas through infrastructure development and economic incentives. In addition, we recommend that the government and other industry organizations widely publicize the dangers of solid fuels and the health benefits of cleaner fuels through traditional and electric media and stimulate the use of cleaner fuels among middle-aged and older adults by increasing their awareness and knowledge of these fuels.

This study has important public health implications and advantages. First, we used nationally representative data to explore the association between cooking fuels, SI, and their transitions, which allows our study to be representative of the Chinese context and provide a scientific reference for the implementation of clean fuel programs. In particular, our study found that even among participants who already had a single SI, the risk of future conversion to DSI may be reduced if they are able to convert solid fuels to clean fuels. This has important implications for reducing the disease burden caused by SI because it is obvious that the severity and cost of DSI are significantly higher than those of a single SI. Second, our study provides new evidence for the early intervention and prevention of SI from the perspective of cooking fuels. Third, we included a series of covariates and performed sensitivity analyses, which made the associations more robust. In particular, we further adjusted for outdoor environmental factors in our sensitivity analysis, and the results indicated that HAP due to cooking fuels was a risk factor for SI, independent of outdoor air pollution.

This study has several limitations. First, we did not include secondhand smoke and fuel use for heating or lighting in the HAP because of a lack of data. The expansion of the implications from the results of this study is expected with further inclusion of additional HAP variables, rather than under the current limitation of the data volume. Second, it is important to note that this study did not explore the association between different types of solid fuels and worsening SI transitions because of China’s vast regional scope and the large number of solid fuel types in different regions, making it difficult to disaggregate them. A more detailed collection of solid fuel types is necessary in the future, which could help further elucidate which solid fuels are more harmful to worsening SI transitions. Third, we used self-reported vision and hearing conditions to evaluate SI, which may have introduced measurement bias. In the future, it will be necessary to use objective instruments to truly measure individuals’ SI and further validate the findings of this study. Fourth, because we excluded participants who lacked data on the main variables, selection bias may have been introduced. Finally, we did not include genetic factors, occupation, income, or biomarkers as covariates due to data limitations. In addition, other confounding factors were not included in the analysis, which may have contributed to residual confounding.

## Conclusions

Solid fuel use was associated with a higher risk of worsening SI transitions in middle-aged and older Chinese adults. Converting the type of cooking fuel from solid to clean fuels may reduce the risk of worsening SI transitions. Tailored clean fuel intervention should be implemented to prevent SI and hence to reduce the burden of SI-related disability.

### Electronic supplementary material

Below is the link to the electronic supplementary material.

Supplementary Material 1

## Data Availability

The data of this study can be obtained on the official website of CHARLS (http://charls.pku.edu.cn/).

## Abbreviations

HAP: Household air pollution
SI: sensory impairments
CHARLS: China Health and Retirement Longitudinal Study
DSI: Dual sensory impairments
HR: Hazard ratio
CI: Confidence interval
VI: Vision impairment
HI: Hearing impairment
PM: Particulate matter
LMICs: Low- and middle-income countries

## References

[1] Li Q, Wang S, Milot E, Bergeron P, Ferrucci L, Fried LP, Cohen AA (2015). Homeostatic dysregulation proceeds in parallel in multiple physiological systems. Aging Cell.

[2] Fischer ME, Cruickshanks KJ, Schubert CR, Pinto AA, Carlsson CM, Klein BEK, Klein R, Tweed TS (2016). Age-related sensory impairments and risk of cognitive impairment. J Am Geriatr Soc.

[3] World Health O. World report on vision. 2019.

[4] Organization WH. World report on hearing. World Health Organization; 2021.

[5] Wang Q, Zhang S, Wang Y, Zhao D, Zhou C (2022). Dual sensory impairment as a predictor of loneliness and isolation in older adults: National Cohort Study. JMIR Public Health and Surveillance.

[6] Fuller SD, Mudie LI, Siordia C, Swenor BK, Friedman DS (2018). Nationwide Prevalence of Self-reported serious sensory impairments and their associations with Self-reported cognitive and functional difficulties. Ophthalmology.

[7] Hwang PH, Longstreth WT, Thielke SM, Francis CE, Carone M, Kuller LH, Fitzpatrick AL (2022). Longitudinal changes in hearing and visual impairments and risk of dementia in older adults in the United States. JAMA Netw open.

[8] Fisher D, Li CM, Chiu MS, Themann CL, Petersen H, Jónasson F, Jónsson PV, Sverrisdottir JE, Garcia M, Harris TB (2014). Impairments in hearing and vision impact on mortality in older people: the AGES-Reykjavik Study. Age Ageing.

[9] Liljas AE, Wannamethee SG, Whincup PH, Papacosta O, Walters K, Iliffe S, Lennon LT, Carvalho LA, Ramsay SE (2016). Socio-demographic characteristics, lifestyle factors and burden of morbidity associated with self-reported hearing and vision impairments in older British community-dwelling men: a cross-sectional study. J Public Health.

[10] Hämäläinen A, Pichora-Fuller MK, Wittich W, Phillips NA, Mick P (2021). Self-report measures of hearing and vision in older adults participating in the Canadian longitudinal study of aging are explained by behavioral sensory measures, demographic, and social factors. Ear Hear.

[11] WHO. WHO guidelines for indoor air quality: household fuel combustion. World Health Organization; 2014. 25577935

[12] Bruce N, Perez-Padilla R, Albalak R (2000). Indoor air pollution in developing countries: a major environmental and public health challenge. Bull World Health Organ.

[13] Frostad JJ, Nguyen QP, Baumann MM, Blacker BF, Marczak LB, Deshpande A, Wiens KE, LeGrand KE, Johnson KB, Abbasi-Kangevari M (2022). Mapping development and health effects of cooking with solid fuels in low-income and middle-income countries, 2000–18: a geospatial modelling study. The Lancet Global Health.

[14] Saenz JL, Adar SD, Zhang YS, Wilkens J, Chattopadhyay A, Lee J, Wong R (2021). Household use of polluting cooking fuels and late-life cognitive function: a harmonized analysis of India, Mexico, and China. Environ Int.

[15] Lee KK, Bing R, Kiang J, Bashir S, Spath N, Stelzle D, Mortimer K, Bularga A, Doudesis D, Joshi SS (2020). Adverse health effects associated with household air pollution: a systematic review, meta-analysis, and burden estimation study. The Lancet Global Health.

[16] Niu X, Jones T, BéruBé K, Chuang HC, Sun J, Ho KF (2021). The oxidative capacity of indoor source combustion derived particulate matter and resulting respiratory toxicity. Sci Total Environ.

[17] Chester J, Johnston E, Walker D, Jones M, Ionescu CM, Wagle SR, Kovacevic B, Brown D, Mikov M, Mooranian A et al. A Review on Recent Advancement on Age-Related Hearing Loss: The Applications of Nanotechnology, Drug Pharmacology, and Biotechnology. *Pharmaceutics* 2021, 13(7). 10.3390/pharmaceutics13071041 PMC8309044 34371732

[18] Macchioni L, Chiasserini D, Mezzasoma L, Davidescu M, Orvietani PL, Fettucciari K, Salviati L, Cellini B, Bellezza I. Crosstalk between long-term sublethal oxidative stress and detrimental inflammation as potential drivers for age-related retinal degeneration. Antioxid (Basel Switzerland) 2020, 10(1). 10.3390/antiox10010025 PMC7823845 33383836

[19] Ju MJ, Park SK, Kim S-Y, Choi Y-H (2022). Long-term exposure to ambient air pollutants and hearing loss in Korean adults. Sci Total Environ.

[20] Yang B-Y, Guo Y, Zou Z, Gui Z, Bao W-W, Hu L-W, Chen G, Jing J, Ma J, Li S (2021). Exposure to ambient air pollution and visual impairment in children: a nationwide cross-sectional study in China. J Hazard Mater.

[21] Chowdhury S, Pillarisetti A, Oberholzer A, Jetter J, Mitchell J, Cappuccilli E, Aamaas B, Aunan K, Pozzer A, Alexander D. A global review of the state of the evidence of household air pollution’s contribution to ambient fine particulate matter and their related health impacts. Environ Int 2023:107835. 10.1016/j.envint.2023.107835 PMC10378453 36857905

[22] Zhou Y, Xu M, Ke P, Di H, Gan Y, Feng J, Meng X, Su C, Tian Q, Lu Z (2023). Association of biomass fuel use with the risk of vision impairment among Chinese older adults: a cohort study. Environ Sci Pollut Res.

[23] Islam S, Upadhyay AK, Mohanty SK, Pedgaonkar SP, Maurer J, O’Donnell O (2022). Use of unclean cooking fuels and visual impairment of older adults in India: a nationally representative population-based study. Environ Int.

[24] Liu T, Cao L, Lv P, Bai S (2022). Associations between household solid fuel use and hearing loss in a Chinese population: a population-based prospective cohort study. Ecotoxicol Environ Saf.

[25] Zhang Y, Ge M, Zhao W, Liu Y, Xia X, Hou L, Dong B (2020). Sensory impairment and all-cause Mortality among the Oldest-Old: findings from the Chinese longitudinal healthy longevity survey (CLHLS). J Nutr Health Aging.

[26] Zhao YH, Hu YS, Smith JP, Strauss J, Yang GH (2014). Cohort Profile: the China Health and Retirement Longitudinal Study (CHARLS). Int J Epidemiol.

[27] Li X, Duan C, Chen Q, Xiao J, Zhang J (2023). Associations between cooking fuels and hypertension prevalence in Chinese adults: a prospective cohort analysis focusing on fuel transitioning. Environ Int.

[28] Rong H, Lai X, Jing R, Wang X, Fang H, Mahmoudi E (2020). Association of sensory impairments with Cognitive decline and Depression among older adults in China. JAMA Netw open.

[29] Maharani A, Dawes P, Nazroo J, Tampubolon G, Pendleton N (2020). Associations between Self-reported sensory impairment and risk of Cognitive decline and impairment in the Health and Retirement Study Cohort. The Journals of Gerontology Series B Psychological Sciences and Social Sciences.

[30] Bikbov MM, Kazakbaeva GM, Rakhimova EM, Rusakova IA, Fakhretdinova AA, Tuliakova AM, Panda-Jonas S, Gilmanshin TR, Zainullin RM, Bolshakova NI (2021). Prevalence factors Associated with Vision impairment and blindness among individuals 85 years and older in Russia. JAMA Netw open.

[31] Bovo R, Ciorba A, Martini A (2011). Environmental and genetic factors in age-related hearing impairment. Aging Clin Exp Res.

[32] Ajmani GS, Suh HH, Pinto JM (2016). Effects of Ambient Air Pollution exposure on olfaction: a review. Environ Health Perspect.

[33] Ghio AJ, Carraway MS, Madden MC (2012). Composition of air pollution particles and oxidative stress in cells, tissues, and living systems. J Toxicol Environ Health Part B.

[34] Hirota JA, Hirota SA, Warner SM, Stefanowicz D, Shaheen F, Beck PL, MacDonald JA, Hackett T-L, Sin DD, Van Eeden S (2012). The airway epithelium nucleotide-binding domain and leucine-rich repeat protein 3 inflammasome is activated by urban particulate matter. J Allergy Clin Immunol.

[35] Glencross DA, Ho T-R, Camiña N, Hawrylowicz CM, Pfeffer PE (2020). Air pollution and its effects on the immune system. Free Radic Biol Med.

[36] Olivetto E, Simoni E, Guaran V, Astolfi L, Martini A (2015). Sensorineural hearing loss and ischemic injury: development of animal models to assess vascular and oxidative effects. Hear Res.

[37] Chang K-H, Tsai SC-S, Lee C-Y, Chou R-H, Fan H-C, Lin FC-F, Lin C-L, Hsu Y-C (2020). Increased risk of sensorineural hearing loss as a result of exposure to air pollution. Int J Environ Res Public Health.

[38] Wei C-C, Lin H-J, Lim Y-P, Chen C-S, Chang C-Y, Lin C-J, Chen JJ-Y, Tien P-T, Lin C-L, Wan L (2019). PM2. 5 and NOx exposure promote myopia: clinical evidence and experimental proof. Environ Pollut.

[39] Gong J, Wang G, Wang Y, Chen X, Chen Y, Meng Q, Yang P, Yao Y, Zhao Y (2022). Nowcasting and forecasting the care needs of the older population in China: analysis of data from the China Health and Retirement Longitudinal Study (CHARLS). The Lancet Public Health.

[40] Pu F, Li C, Zhang X, Cao X, Yang Z, Hu Y, Xu X, Ma Y, Hu K, Liu Z (2023). Transition of cooking fuel types and mortality risk in China, 1991–2015. Sci Total Environ.

[41] Meng W, Zhong Q, Chen Y, Shen H, Yun X, Smith KR, Li B, Liu J, Wang X, Ma J. Energy and air pollution benefits of household fuel policies in northern China. *Proceedings of the national academy of sciences* 2019, 116(34):16773–16780. 10.1073/pnas.1904182116 PMC6708357 31383761

[42] Li Y, Yuan X, Tang Y, Wang Q, Ma Q, Mu R, Fu J, Hong J, Kellett J, Zuo J (2020). Integrated assessment of the environmental and economic effects of coal-to-gas conversion project in rural areas of northern China. Environ Sci Pollut Res.

