# Supplementary material for: Associations between cooking fuel use, its transitions, and worsening sensory impairments among Chinese middle-aged and older adults: a cohort study
Source: BMC Geriatr. 2024 Mar 27;24:288. doi: 10.1186/s12877-024-04746-3 (PMC10976684; doi:10.1186/s12877-024-04746-3)
Supplement: Supplementary file 1 — Supplementary Material 1 [file 12877_2024_4746_MOESM1_ESM.docx]

## Associations between cooking fuel use, its transitions, and worsening sensory impairments among Chinese middle-aged and older adults: a cohort study

## Supplementary Materials

1. **The measurement of cognitive function**

In CHARLS, the measurement of cognitive function includes four parts: orientation (5 points), memory (20 points), computation (5 points), and drawing ability (1 point) ^1^. The sum of the four scores is the total score of cognitive function. The total score ranges from 0 to 31, while the higher the score, the healthier the cognitive functionality. Since there is no clear cut-off value for mild cognitive impairment in CHARLS, we selected “at least 1 standard deviation (SD) below the aged standard” as the cut-off value according to Aging-associated cognitive decline (AACD) diagnostic criteria of Working Party of the International Psychogeriatric Association ^2^. Participants were divided into groups according to every 5 years of age, and those with a cognitive function score of less than a mean of -1 SD in each group were classified into mild cognitive impairment.

1. **The measurement of PM_2.5_**

To protect the privacy of the participants, CHARLS did not give the specific location of each participant's residence, but only the city where they are located is given. Therefore, the PM_2.5_ measured in this study are city-level data. PM_2.5_ data were obtained from the global surface PM _2.5_ dataset (resolution of 0.01° × 0.01°) established by the Atmospheric Composition Analysis Group to estimate the average PM_2.5_ concentration^3^ in the year prior to the survey in the participant's city. We calculated the city-level average PM_2.5_ concentrations for the first 12 months of the survey month in 2011 based on CHARLS administrative boundary data from Global/Regional Estimates (V5.GL.02).

## Supplementary references

1. Luo Y, Zhong Y, Pang L, Zhao Y, Liang R, Zheng X. The effects of indoor air pollution from solid fuel use on cognitive function among middle-aged and older population in China. Sci Total Environ. 2021; 754:142460.

2. Levy R. Aging-associated cognitive decline. Working Party of the International Psychogeriatric Association in collaboration with the World Health Organization. International psychogeriatrics. 1994; 6:63-8.

3. Hammer MS, van Donkelaar A, Li C, Lyapustin A, Sayer AM, Hsu NC, et al. Global Estimates and Long-Term Trends of Fine Particulate Matter Concentrations (1998–2018). Environmental Science & Technology. 2020; 54:7879-90.
